# Family level variation in *Wolbachia*-mediated dengue virus blocking in *Aedes aegypti*

**DOI:** 10.1186/s13071-017-2589-3

**Published:** 2017-12-28

**Authors:** Gerard Terradas, Scott L. Allen, Stephen F. Chenoweth, Elizabeth A. McGraw

**Affiliations:** 1 School of Biological Sciences, Monash University, Clayton, Melbourne, VIC Australia; 2 School of Biological Sciences, The University of Queensland, QLD, St. Lucia, Australia

**Keywords:** *Aedes*, Genetic variation, *Wolbachia*, Dengue virus, Evolution

## Abstract

**Background:**

The mosquito vector *Aedes aegypti* is responsible for transmitting a range of arboviruses including dengue (DENV) and Zika (ZIKV). The global reach of these viruses is increasing due to an expansion of the mosquito’s geographic range and increasing urbanization and human travel. Vector control remains the primary means for limiting these diseases. *Wolbachia pipientis* is an endosymbiotic bacterium of insects that has the ability to block the replication of pathogens, including flaviviruses such as DENV or ZIKV, inside the body of the vector. A strain of *Wolbachia* called *w*Mel is currently being released into wild mosquito populations to test its potential to limit virus transmission to humans. The mechanism that underpins the virus blocking effect, however, remains elusive.

**Methods:**

We used a modified full-sib breeding design in conjunction with vector competence assays in wildtype and *w*Mel-infected *Aedes aegypti* collected from the field. All individuals were injected with DENV-2 intrathoracically at 5–6 days of age. Tissues were dissected 7 days post-infection to allow quantification of DENV and *Wolbachia* loads.

**Results:**

We show the first evidence of family level variation in *Wolbachia*-mediated blocking in mosquitoes. This variation may stem from either genetic contributions from the mosquito and *Wolbachia* genomes or environmental influences on *Wolbachia*. In these families, we also tested for correlations between strength of blocking and expression level for several insect immunity genes with possible roles in blocking, identifying two genes of interest (*AGO2* and *SCP-2*).

**Conclusions:**

In this study we show variation in *Wolbachia*-mediated DENV blocking in *Aedes aegypti* that may arise from genetic contributions and environmental influences on the mosquito-*Wolbachia* association. This suggests that *Wolbachia*-mediated blocking may have the ability to evolve through time or be expressed differentially across environments. The long-term efficacy of *Wolbachia* in the field will be dependent on the stability of blocking. Understanding the mechanism of blocking will be necessary for successful development of strategies that counter the emergence of evolved resistance or variation in its expression under diverse field conditions.

**Electronic supplementary material:**

The online version of this article (10.1186/s13071-017-2589-3) contains supplementary material, which is available to authorized users.

## Background

*Wolbachia pipientis* is an insect endosymbiont capable of manipulating host reproductive success via different mechanisms, the primary and most studied being cytoplasmic incompatibility (CI) [1]. CI gives *Wolbachia*-infected females a reproductive advantage and because the symbiont is maternally transmitted, the bacterium spreads rapidly through uninfected populations. *Wolbachia* also reduces susceptibility of their hosts to a range of pathogens, including viruses, other bacteria, nematodes, fungi and the malaria parasite [2–6]. The traits of CI and *Wolbachia*-mediated pathogen blocking together form the basis of emerging strategies to use *Wolbachia* as an agent of biocontrol against vector-borne diseases [7]. Though present in an estimated 40% of all insect species [8], *Wolbachia* is naturally absent in the main dengue vector, *Ae. aegypti*. However, stably inherited *Wolbachia* infections with a range of strains (*w*Mel & *w*MelPop originally from *Drosophila melanogaster* and *w*AlbB from *Aedes albopictus*) have been created in the mosquito using microinjection techniques [9–11]. Adult *Ae. aegypti* mosquitoes infected with *w*Mel [12] and *w*AlB [9] are currently being released into the wild to test the ability of *Wolbachia* to spread and to limit human disease [13].

Natural *Ae. aegypti* populations vary in their susceptibilities to dengue virus (DENV) [14–18] and laboratory-based breeding experiments have demonstrated substantial contribution of the mosquito genome to variation in susceptibility often through the innate immune response [17, 19–21]. When *Wolbachia* infection is present, pathogen blocking is exhibited by reductions in viral infection rates, loads and transmissibility [5, 22–25] beyond the wildtype host’s natural antiviral mechanisms. *Wolbachia*’s presence throughout the body of the mosquito [5, 11] provides numerous opportunities for the symbiont to interfere with the successful colonization and replication of viruses. Inside cells, *Wolbachia* lives within a vacuole of host origin [26, 27] utilizing transporters to feed off host resources like amino acids that its incomplete genome cannot synthesize [28, 29], and communicating with the extracellular environment using a Type IV secretion system [30, 31]. *Wolbachia*-mediated phenotypes including pathogen blocking must therefore, by necessity, be enacted via host physiologies and across host membranes. We would therefore predict that variation in the mosquito genome is likely to play a role in *Wolbachia*-mediated blocking.

It is unclear whether the *Wolbachia* genome evolves fast enough to be a substantial contributor to variation in the trait. Each generation the population of inherited symbiont experiences a bottleneck at the point of transmission via the embryo [32, 33] and there is little opportunity to exchange genes with diverse *Wolbachia* strains in the intracellular environment [34]. In the case of stable transinfection of the *w*MelPop strain into *Ae. aegypti* no new substitutions were witnessed in the symbiont genome in the 4-year period post-introduction [35]. Changes have been demonstrated however in a *Wolbachia* strain’s effects on *Drosophila simulans* over a longer timeframe [36, 37].

Understanding the mechanistic underpinning of the blocking trait, and in particular its complexity, is necessary to assess the role that genetic variation and evolution may play in shaping the trait’s expression in the field. Various theories have arisen with regard to mechanism [38]. The first theory suggested that *Wolbachia* may “prime” or activate the host immune response, leading to a heightened ability to limit the growth and replication of subsequent infections with pathogens [4, 22, 39–41]. While there is growing evidence that immune priming may provide blocking against bacterial pathogens [42], innate immunity may only offer a small boost in viral blocking [43, 44]. A second set of theories relate to competition for resources between *Wolbachia* and incoming pathogens. The resources have included intracellular space [5, 45], lipids [26, 46, 47] and nitrogen [48]. Nitrogen may serve as a primary source of energy for *Wolbachia* [48] and *Wolbachia*’s modulation of lipid profiles in insect cells may create an environment that is antagonistic toward viral replication [47]. A third set of studies suggests that *Wolbachia* may manipulate expression of host genes that control viruses via microRNAs [49–51]. Most recently, several studies have indicated that *Wolbachia* infection may alter fundamental structures [52] or environments in the host cell [53] that prevent viral replication immediately after entry into cells. A trend that is compatible with all of the above mechanistic explanations for blocking is that higher *Wolbachia* loads are associated with stronger blocking [11, 54–56].

As the *Wolbachia* genome is intimately tied to that of the host through maternal inheritance, it is difficult to tease apart the independent genetic contributions of the partners to the trait [43]. In the ideal experimental scenario, we could partition the relative contribution of the mosquito and *Wolbachia* genomes as well as the role of the environment in determining variation in DENV blocking. Such traditional quantitative breeding approaches would require the same mosquito families to be studied with and without *Wolbachia* infection. As transinfection of mosquitoes often requires injection of thousands of individuals to achieve success [10] and removal of *Wolbachia* by antibiotic treatment takes multiple generations [57], the ideal experiment cannot be done. Instead, we have used a modified full-sib breeding design approach to assess family level variation in *Wolbachia-*mediated blocking in a population of Australian *Ae. aegypti*. By examining the same trait in parallel in *Wolbachia*-free mosquito families we were also able to demonstrate the additional contribution (both genotypic and environmental) of *Wolbachia* infection to the variance of dengue virus load. We then used families exhibiting the phenotypic extremes in DENV blocking to screen four candidate mosquito genes for correlations in expression that would be suggestive of a functional role in blocking. We used qPCR gene screening as a proof of principle to see whether we could detect relationships between gene expression behavior and strength of *Wolbachia*-mediated blocking. The candidates tested were selected because they had previously been shown to be modulated by *Wolbachia* and also play a role in DENV infections.

## Results

### DENV load in head tissue by family

**Fig. 1:**
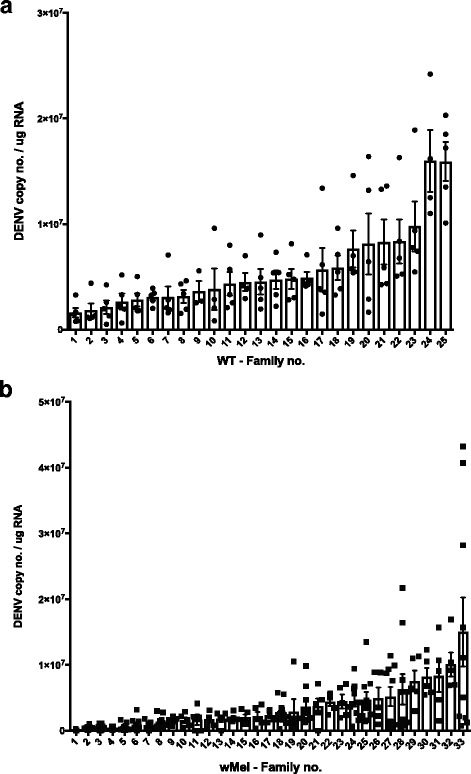
Head DENV load. WT (**a**) and *w*Mel-infected (**b**) families with mean and SEM depicted

Breeding in a modified full-sib [58, 59] framework yielded 25 wildtype and 33 *w*Mel-infected *Ae. aegypti* families with sufficient offspring for injections. For each family 5 to 30 females were injected with DENV-2 and then their midgut, head and carcass (representing the rest of the body) were dissected at 7–8 days post-infection (dpi). After RNA extraction of 5+ individual heads per family, DENV-2 load was quantified via RT-qPCR. Head DENV loads have been commonly used as a proxy for dissemination of the virus [11, 60, 61] and so we used them to rank families (Fig. 1). Carcasses from the selected individuals were then used to test for *Wolbachia* loads and gene expression analyses. All individuals for both WT and *w*Mel lines were infected given the use of intrathoracic injection that bypasses the midgut infection barrier and allows the virus to disseminate freely. As expected, due to the action of blocking, DENV loads were lower in *w*Mel families compared to WT (*t* = 31.94, *df* = 340, *P* < 0.0001). Heritabililties for DENV load were high and significantly greater than zero for each line; WT [H^2^ = 0.95 (0.54–1.29), LRT: *χ* ^2^ = 38.4, *P* = 5.76 × 10^−10^] and *w*Mel [H^2^ = 0.85 (0.51–1.23), LRT: *χ* ^2^ = 70.0, *P* = 1.11 × 10^−6^]. Given the maternal inheritance of *Wolbachia*, the latter estimate will be highly inflated, suggesting greater similarity across families due to shared environmental variation and linkage of host and *Wolbachia* genomes. The slightly lower heritability may suggest that *Wolbachia* infection and its interaction with the host is introducing additional variation compared to the simple system involving the vector and virus alone.

### DENV load in carcass tissue by family

**Fig. 2:**
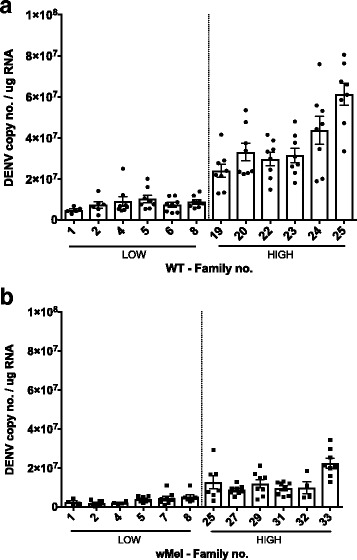
Carcass DENV load. Differences in DENV load in carcass of families previously classified as High and Low by head tissue (**a**) WT and (**b**) *w*Mel-infected individuals, mean and SEM

To determine if the differences seen in DENV loads for the heads correspond to similar differences in carcasses, RNA extractions were performed on carcasses from individuals previously classified as extreme families (Low and High, Fig. 1). Carcass DENV loads mostly recapitulated the patterns seen in heads (Fig. 2, Additional file 1: Figure S1) and for each treatment we selected 6 families that were most concordant for subsequent analysis (Fig. 2). A generalized nested mixed model was used to test for differences between low and high clusters. *Wolbachia* infection status (*F*_(1)_ = 15.32, *P* = 0.001), DENV load (*F*_(1)_ = 26.39, *P* < 0.001) as well as the interaction between these two main factors and family (*F*_(21)_ = 9.47, *P* < 0.001) were significant. The significant interaction is due to the higher range of DENV loads in WT families, given pathogen blocking in the *w*Mel line.

### *Wolbachia* correlation to DENV titres

**Fig. 3:**
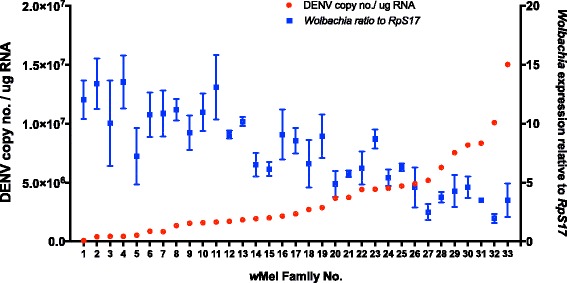
*Wolbachia* loads determine pathogen protection. Red circles show family means for DENV load (left axis). Blue squares depict mean *Wolbachia* counts relative to *RpS17,* with SEM (right axis)

To assess the variability of *Wolbachia* densities amongst families as well as a possible *Wolbachia*-based determination of DENV loads, gDNA was extracted from 3 individual carcasses per family and *Wolbachia* levels were checked using qPCR. As mean *Wolbachia* densities rise in families, DENV loads decline (Fig. 3). This negative correlation was significant (Additional file 1: Figure S2; *r* = 0.546, *P* < 0.0001) and may indicate greater protection against DENV dissemination in the carcass in response to *Wolbachia*. Virus infection did not have an effect on *Wolbachia* loads (Additional file 1: Figure S3, *P* = 0.16).

### Candidate gene expression: immunity

**Fig. 4:**
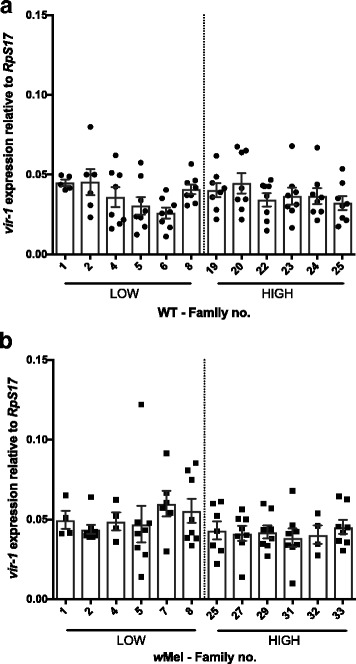
*vir-1* expression in families classified as High and Low DENV. Graphs show the expression of *vir-1* relative to the housekeeping gene *RpS17* in (**a**) WT individuals, filled circle and (**b**) *w*Mel-infected individuals, filled square. Means with SEM (*n* = 8)

**Fig. 5:**
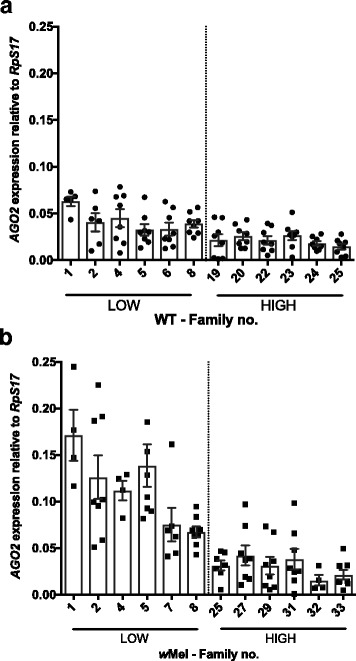
*AGO*2 expression in families classified as High and Low DENV. Graphs show the expression of *AGO*2 relative to the housekeeping gene *RpS17* in (**a**) WT individuals, filled circle and (**b**) *w*Mel-infected individuals, filled square. Means with SEM (*n* = 8)

Having confirmed that the *w*Mel strain reduces DENV replication at an individual and population level, we then used our families with extreme blocking phenotypes to test for associations with expression of immunity genes with potential roles in blocking (Figs. 4 and 5). We focused on *vir-1* and *AGO2*, genes that represent the two major antiviral pathways in mosquitoes, JAK/STAT and RNAi, respectively [62]. The latter gene has been shown to play a minor role in DENV blocking in mosquito cells [44]. Gene expression was analyzed using a generalized mixed model with the random variable ‘Family’ nested with *Wolbachia* × DENV load, with *Wolbachia* and DENV load as fixed factors. The effect of *Wolbachia* infection was significant (Fig. 4; *F*_(1)_ = 12.83, *P* = 0.002), causing upregulation in the expression of *vir-1*. However, *vir-1* expression was not associated with DENV load/family (Fig. 4; *F*_(1)_ = 3.1, *P* = 0.091). There was also no significant interaction between the two main factors (*F*_(21)_ = 1.05, *P* = 0.412, Additional file 1: Figure S4a). These results suggest that while *vir-1* levels may be important for DENV control in the mosquito they do not explain variation in the blocking trait in *Wolbachia*-infected mosquitoes at least at the time point surveyed post-infection.

The same mixed effects model was applied to test for differences in *argonaute-2* (*AGO2*) gene expression levels. The effect of *Wolbachia* was significant (Fig. 5; *F*_(1)_ = 16.72, *P* = 0.001), leading to heightened expression of the gene. We also detected a significant effect of DENV load/family (Fig. 5; *F*_(1)_ = 27.62, *P* < 0.001), demonstrating higher expression of the gene in Low DENV load families. The interaction was also significant (*F*_(21)_ = 5.26, *P* < 0.001), showing that the differences between High and Low DENV loads in *AGO2* expression are greater in *w*Mel-infected mosquitoes than in WT (Additional file 1: Figure S4b). In WT families, gene expression decreases as DENV titres increase. The same is true for *w*Mel-infected families, but with an even greater disparity between Low and High families.

### Candidate gene expression: host factor competition

We also examined how genes involved in intracellular lipid transport (*Sterol carrier protein 2*, *SCP-2*) and nitric oxide biosynthesis (*Nitric oxide synthase*, *NOS*) are differentially expressed for each cell line and cluster. These genes have previously been proposed as not only important for lipid distribution or nitrogen production but also to be critical for DENV infection in *Ae. aegypti* [63, 64]. The bacterium and the virus are hypothetically competing for host nutrients and thus providing the host with a *Wolbachia*-mediated blocking phenotype.

**Fig. 6:**
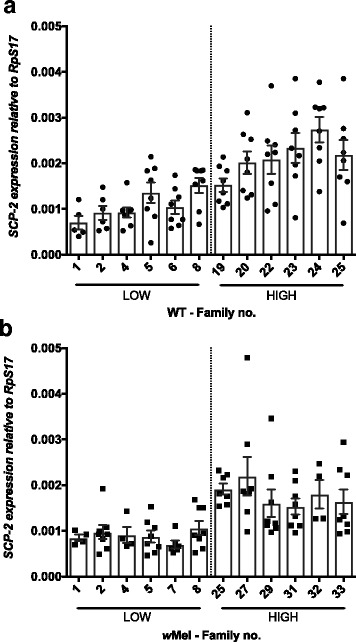
*SCP-2* expression in families classified as High and Low DENV. Graphs show the expression of *SCP-2* relative to the housekeeping gene *RpS17* in (**a**) WT individuals, filled circle and (**b**) *w*Mel-infected individuals, filled square. Means with SEM (*n* = 8)

The effect of *Wolbachia* infection on *SCP-2* expression was significant (Fig. 6; *F*_(1)_ = 5.01, *P* = 0.035), with *SCP-2* expression slightly down regulated in *w*Mel mosquitoes relative to WT. We also see a significant DENV load effect on gene expression (Fig. 6; *F*_(1)_ = 64.91, *P* < 0.001). In this case, contrary to what we see in *AGO2* expression, *SCP-2* levels are higher in those individuals clustered into High DENV Load for both WT and *w*Mel-infected mosquitoes and hence the interaction was not significant (Additional file 1: Figure S4c; *F*_(21)_ = 1.5, *P* = 0.087). This suggests that while *SCP-2* may be a contributing factor to viral success in mosquitoes, its expression is not associated with variation in *w*Mel-mediated blocking.

**Fig. 7:**
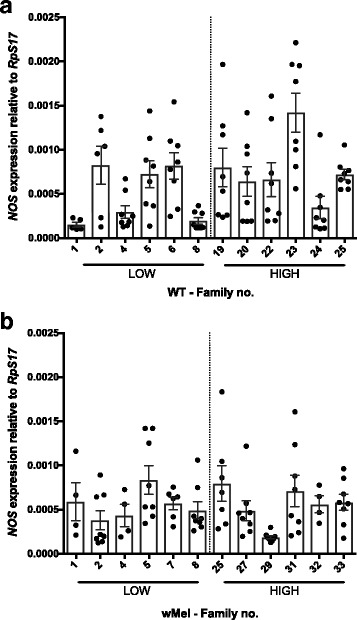
*NOS* expression in families classified as High and Low DENV. Graphs show the expression of *NOS* relative to the housekeeping gene *RpS17* in (**a**) WT individuals, filled circles and (**b**) *w*Mel-infected individuals, filled squares. Means with SEM (*n* = 8)

For *NOS*, neither *Wolbachia* infection (Fig. 7; *F*_(1)_ = 0.48, *P* = 0.491) nor DENV load (Fig. 7, *F*_(1)_ = 1.3, *P* = 0.267) had an effect on the gene’s expression. However, the interaction was significant (Fig. 7, *F*_(21)_ = 3.73, *P* < 0.001). The nature of the interaction is difficult to interpret given the high level of variation in expression between families particularly for the WT line (Fig. 7, Additional file 1: Figure S4d). These data would suggest that *NOS* expression is unlikely to be associated with *Wolbachia*-mediated blocking.

## Discussion

In this study we aimed to measure family level variation present in the *Wolbachia*-mediated pathogen blocking trait in mosquitoes infected with the *w*Mel strain. To do so, we performed a modified full-sib breeding design that allowed us limit the contribution of environmental variation to the trait but not completely remove it given the maternal inheritance in *Wolbachia*. We were then able to use families representing the phenotypic extremes in blocking to test for correlations in gene expression for a number of candidate genes for the basis of the trait.

The experiments demonstrate that there is greater variation in DENV loads in the *w*Mel-infected mosquitoes compared to wildtype mosquitoes. The DENV loads in extreme families of *w*Mel mosquitoes spanned 45-fold compared to the 5-fold difference seen for WT. DENV infection success in WT mosquitoes is highly influenced by genotype:genotype interactions between mosquito and virus [17, 60, 65]. The greater variation in *w*Mel mosquitoes may stem from contributions from the *Wolbachia* genome, as well environmental influences on the symbiont, confounded with family. Variation in pathogen blocking due to differences in *Wolbachia* strains has been demonstrated previously in *Drosophila* [66].

Studies that have examined phenotypic variation in blocking in both *Ae. aegypti* and *Drosophila* also show correlations between *Wolbachia* density and the strength of pathogen blocking [5, 11, 54, 66, 67]. Therefore, after determining the blocking phenotype in the families, we also examined the variation in *Wolbachia* load for the *w*Mel-infected population. We observed a high degree of variability in *Wolbachia* levels among families. Within families this measure will be confounded or inflated by *Wolbachia*’s near perfect mode of vertical transmission. *Wolbachia* loads in the carcass also correlated with pathogen-blocking ability as predicted. While recent work from our group suggested that *Wolbachia* loads in particular tissues may not determine blocking strength [68], our study reaffirms the relationship for total *Wolbachia* loads.

*Wolbachia* is currently being assessed for its capacity to limit dengue virus transmission from mosquitoes to humans in the field [12, 69, 70]. The long-term efficacy of *Wolbachia* is not only reliant on the effective spread of the symbiont in the population but also dependent on the stability of expression of the blocking trait. Understanding how much variation and in particular genetic variation there is for blocking and *Wolbachia* load is critical. This is because populations can only adapt and change if there is genetic variation present for the trait of interest [71]. Blocking may be expected to vary across genetically diverse mosquito populations, in response to diverse viruses, over a range of environmental conditions and with sufficient co-evolutionary time in response to diverging *Wolbachia* strains. Given genetic variation in both host and symbiont we may be able to predict the outcome of co-evolutionary pressures. Interestingly, during a two-year period surveyed after release of the *Wolbachia* strain *w*Mel into wild populations, neither host longevity nor DENV blocking showed evidence of change [23, 72].

First, if high densities of *Wolbachia* confer better blocking but those densities are detrimental to the host, we may expect selection for reduced loads or lowered maternal transmission rates. The detriment to the host may come from the costs of producing an immune response [73] or supporting a symbiont with complex metabolic needs [46, 74]. Additionally, there may be direct effects of damage on infected cells and tissues. The extreme form of this is demonstrated by the *w*MelPop strain [75] that overgrows inside host cells and causes cell lysis, the result being shortened lifespan. While the other strains of *Wolbachia* being developed for biocontrol, *w*Mel and *w*AlB, do not appear to cause cellular destruction, they still induce an immune response and spend a portion of their cellular resources on *Wolbachia* [22, 39]. In the laboratory, these effects do not appear to have substantial impacts on the insect’s reproductive output [72, 76]. Lastly, modelling has demonstrated that even with some negative fitness costs, the high maternal transmission and CI of *Wolbachia* will help it remain in populations [72].

Secondly, the impact of viral and other infectious agents on the insect may select for stronger blocking. Flaviviral infections can result in fitness costs for the mosquito; in the case of DENV, both reduced fertility and lifespan are affected [77]. *Wolbachia*-mediated blocking would attenuate these potential fitness costs associated to a high viral infection, as infection rates are lower in *Wolbachia*-infected mosquitoes and for those that become infected, severity is reduced [23]. Therefore, selection pressure for the blocking trait would be greater in areas with a high incidence of DENV and other flaviviruses. Additionally, there may be protection of native viruses [78] although it is unclear what impact these viruses have on host fitness if any. In *D. melanogaster*, the symbiont does not appear to affect native viral diversity [79]. Lastly, *Wolbachia* could protect against systemic bacterial, fungal or other parasitic infections, encountered by insects in the field, the nature of which are very poorly understood.

Using our extreme families with respect to DENV blocking, we were able to test for correlations for several candidate genes for the mechanism of blocking. Gene expression is highly plastic and if the blocking trait was reliant on *Wolbachia*-mediated modulation of some genes, the phenotype of the trait could vary rapidly due to co-evolution between *Wolbachia* and the mosquito [80]. Gene modulation in response to the symbiont is likely to be reduced greatly over time if the differences between novelly and natively infected hosts are predictive. For example, in *Drosophila* with long standing *Wolbachia* associations, the immune response is negligible [81]. We assessed genes involved in the humoral responses (*vir-1* and *AGO2*), intracellular lipid transport (*SCP-2*) and nitrogen production (*NOS*). Interestingly, *AGO2* and *SCP-2* showed a correlation between their levels of expression and DENV load, which reaffirms that they play a role in the viral infection. However, neither are sufficient to explain *Wolbachia*-mediated blocking of DENV infection [44]. The JAK/STAT effector *vir-1* and *NOS* however, did not have patterns of expression related to strength of pathogen blocking trait. These data are in keeping with other studies [39, 53], suggesting that the immune response to *Wolbachia*, particularly present in novelly infected hosts, cannot explain a significant portion of blocking.

Several aspects of the study may limit its interpretation. As detailed above, the inheritance pattern of *Wolbachia* limits our ability to fully partition environmental and genetic variances. It also leads to correlations between DENV and *Wolbachia* loads in families. Regardless of this, the approach was able to limit the contribution of environmental influences by controlled breeding and infection of mosquitoes. Additionally, the approach used viral microinjection to infect mosquitoes due to the constraints of blood-feeding compliance and difficulties with obtaining disseminated infections in *w*Mel-infected mosquitoes due to pathogen blocking. This method will not capture any of the variation in the trait associated with the midgut as it is bypassed by injection. However there is little evidence of strong W*olbachia* loads in the midgut [68] and it is not clear if this tissue contributes heavily to blocking. Also, we tested for DENV load at a single time point post-injection. Blocking phenotypes may vary with time, as would gene expression profiles [82]. It is plausible, for example, that gene expression levels for the candidate genes peak immediately after blood-feeding or exposure to the virus but decrease as soon as infection is established and viral replication promoted. Moreover, we only tested four candidate genes but for those that proved significant, further experiments such as RNAi-based knockdown or other gene modification techniques should be performed in adult mosquitoes to further elicit the contribution of both *AGO2* and *SCP-2* to DENV infections.

## Conclusions

In this study we demonstrated substantial variation in *Wolbachia*-mediated DENV blocking in mosquitoes that may spring from genetic contributions from both partners and environmental influences on *Wolbachia*, not controlled by family breeding. This suggests that the *Wolbachia*-mediated blocking may have the opportunity to evolve through time or be expressed differentially across diverse environments. The long-term efficacy of *Wolbachia* as a biocontrol tool will be dependent on the stability of blocking. We suggest the use of genome wide association studies to identify candidate genes that affect blocking. While the confounding of *Wolbachia* inheritance and environmental factors may lead to higher numbers of false positives, further functional testing using genetic modification would allow the isolation of key loci. Such broad genomic approaches offer the best means for identifying candidate pathways in the mosquito and *Wolbachia* without any a priori assumptions about how blocking might work. Understanding the mechanism of blocking will be necessary for the successful development of strategies [83] to counter the emergence of evolved resistance or variation in its expression under diverse conditions.

## Methods

### Mosquito collection

All *Ae. aegypti* mosquitoes collected from the field were identified by morphology and later checked by qPCR primer detection [12]. Two *Ae. aegypti* mosquito lines were used in this study: wildtype (WT) and *Wolbachia*-infected (*w*Mel). WT are naturally *Wolbachia* free and their eggs were collected outside the Eliminate Dengue *Wolbachia* release zone [12] in greater Cairns, Australia, whereas eggs from the transinfected line *w*Mel were collected from inside the same *Wolbachia* release zone and reared in the lab for 13 generations prior to the start of this study. Both lines were screened for presence/absence of *Wolbachia* infection using the same qPCR methods. At every generation, *w*Mel females were backcrossed to 20% uninfected WT males within 3 generations of the field to limit differences in genetic background while maintaining *Wolbachia* infection [24].

### Mosquito rearing and family design

A modified full-sib [58, 59] breeding design was performed independently in WT and *w*Mel *Ae. aegypti* mosquitoes. After synchronized egg hatching, mosquitoes were reared at a density of ~150 larvae in 30 × 40 × 8 cm trays containing 3 l of RO water. Rearing was performed under controlled conditions of temperature (26 ± 2 °C), humidity (~70%) and photoperiod (12:12, light:dark). Larvae were fed fish food (Tetramin, Melle, Germany). After pupation, males and females were sexed and transferred separately to 30 × 30 × 30 cm cages to allow eclosion at a density of ~450 individuals/cage. Adult mosquitoes were fed a 10% sucrose water diet. Six to eight day-old adult females (P1) were group fed on human volunteers. A total of 250 isofemale pairs containing a male and a blood-fed virgin female were placed in small housings. Eggs laid by isofemales on moist filter paper were collected every 2 days and dried uniformly for short-term storage. We chose families that produced more than 25 eggs that did not suffer from desiccation. F1 individuals from each family were hatched in deoxygenated water and interbred to increase the population number in F2. The experiment was performed using 25 WT and 33 *w*Mel independent families that produced sufficient numbers of eggs.

### Virus

All experiments were carried out with a dengue virus serotype 2 strain (DENV-2, ET300) isolated from human serum collected from patients from East Timor in 2000. The virus was propagated in cell culture as described previously [84] before any experimental use. C6/36 cells were grown in RPMI 1640 media (Life Technologies, Carlsbad, CA, USA) and supplemented with 10% heat-inactivated fetal bovine serum (FBS, Life Technologies), 1% Glutamax (Life Technologies) and 25 mM HEPES (Sigma-Aldrich, St. Louis, MO, USA). Cells were maintained in a non-humidified incubator at 25 °*C. prior* to injection, C6/36 cells were grown to 70–80% confluence and ET300 infective virions were allowed to attach to the cells for 2 h, washed and then maintained in 2% FBS media. Virus was harvested at 7 dpi by collecting the cell culture supernatant before centrifugation at 3200 rpm for 15 min at 4 °C. Viral stocks were stored in individual aliquots at −80 °C until further use and titrated after using plaque assays.

### Intrathoracic microinjections

DENV infected blood was injected to ensure uniformity of dosage that cannot be obtained by blood-feeding. *Aedes aegypti* females were briefly anesthetized with CO_2_ and DENV was injected under a microscope using a pulled glass capillary with a manual microinjector (Nanoject II, Drummond Sci., Broomall, PA, USA). Sixty-nine microlitres of diluted virus stock (~70 DENV pfu) were delivered intrathoracically into every *Ae. aegypti* female. After injection, mosquitoes were maintained under identical initial controlled conditions at 25 °C with 60% relative humidity, 12 h light/dark cycle and feeding on a 10% sucrose solution.

### Dissection of tissues

At 7–8 dpi, females were knocked down via CO_2_ and dissected in 1× phosphate buffered saline (PBS). Head, midguts and carcasses were dissected for 5–15 females per family. Dissecting needles were soaked in 80% ethanol between individual dissections to limit contamination. Different sets of needles were used for WT and *w*Mel dissections. Dissected tissues were immersed in 200 μl of TRIzol (Invitrogen, Carlsbad, CA, USA) in a 1.5 ml tube containing a 3 mm glass bead (Merck KGaA, Darmstadt, Germany). Dissected samples were immediately placed on ice, lysed using a mini-beadbeater (BioSpec Products, Bartlesville, OK, USA), snap frozen and stored at -80 °C until further processing. Any remaining injected mosquitoes per family were collected, frozen and stored at -80 °C as whole insects.

### RNA/DNA extractions

Head and carcass samples were extracted using the manufacturer’s protocol for TRIzol reagent (Invitrogen). Both DNA and RNA phases were collected. RNA was quantified using a Synergy™ MX microplate reader (Biotek, Winooski, VT, USA). All RNA samples were normalized by diluting to an even concentration of 10 ng/μl prior to analysis. Genomic DNA was stored at -80 °C, until subsequent extraction with back extraction buffer (4 M guanidine thiocyanate +50 mM sodium citrate +1 M Tris pH = 8) according to the manufacter’s guidelines for Trizol (Invitrogen).

### DENV qRT-PCR and analysis

All qPCR assays were run on a LightCycler480 Instrument (Roche Applied Science, Basel, Switzerland). One-step quantitative RT-PCR (qRT-PCR) to detect DENV titres was performed using TaqMan Fast Virus 1-step Master Mix (Roche Applied Science) in a total of 10 μl, following manufacturer’s instructions. Standards and samples were run in duplicate. Primer sequences used for DENV detection can be found in Additional file 1: Table S1. DENV qRT-PCR reactions were performed and run as described previously [44]. The number of viral copies present in each sample was evaluated using known standards [5]. The used standards ranged from 10^8^ to 10 DENV fragment copies. The limit of detection was set at 100 copies as the virus was consistently detected at this level. Concentration of DENV in each sample was extrapolated from the standard curve and back calculated to DENV copies/μg of total RNA.

### Analysis of genetic variance

Genetic variance and subsequent broad-sense heritabilities (H^2^) for the focal traits (DENV and *Wolbachia* load) were estimated using a modified full-sib breeding design and the following random effects linear model:

1 $$ {z}_{ij}={f}_i+{\varepsilon}_{ij} $$

where *z*_*ij*_ is the trait value for the *j*th female from the *i*th family, *f*_*i*_ is the random effect of the *i*th family and *ε*_*ij*_ is the unexplained error. To test whether genetic variance was greater than zero, model (1) was compared to a reduced model that had the family term omitted. A likelihood ratio test was constructed where twice the difference in log likelihood between the full and reduced models was contrasted with a Chi-squared distribution with one degree of freedom [85]. All models were fit using SAS version 9.3 (SAS Institute, Cary, NC, USA) separately on the wildtype and *w*Mel-infected groups. Broad-sense heritability was calculated as twice the genetic variance (*σ*_*family*_) divided by the total phenotypic variance (*σ*_*family*_ + *σ*_*error*_).

### Candidate gene expression

All carcass samples were retrotranscribed from RNA to cDNA using the SuperScript III Reverse Transcriptase kit (Invitrogen) containing 12.5 μl of RNA template, 1 μl of random primers (RP, 125 ng/μl), 1 μl of deoxynucleotides (dNTPs, 2.5 mM), dithiothreitol (DTT), 5× buffer and enzyme as per kit instructions, totaling a volume of 20 μl. cDNA synthesis was performed in a C1000 Thermal Cycler (Bio-Rad, Hercules, CA, USA) on the following temperature profile: 5′ at 65 °C followed by 10′ at 25 °C, 50′ at 50 °C, 10′ at 75 °C and kept at 4 °C. Gene expression levels were estimated using the SYBR^®^ Green I Master (Roche Applied Science) with 1 μl of the previously synthesized cDNA, following manufacturer’s instructions. All CT values were normalized to the housekeeping *Ae. aegypti RpS17* gene [86], whose expression was consistent in different samples and mosquito lines. Expression ratios were obtained using the ∆∆Ct method [87]. All primers for candidate genes are listed in Additional file 1: Table S1.

### *Wolbachia* quantification

*Wolbachia* carcass densities were quantified after DNA extraction using a set of *w*Mel-specific primers amplifying for the IS5 repeat element [88]. TaqMan multiplex qPCR was carried out following manufacturer’s protocol (Roche Applied Science). The primers used can be found in Additional file 1: Table S1. *Wolbachia* to *RpS17* housekeeping ratios were calculated using the ∆∆Ct method [87].

### Statistics and data analysis

All qPCR reactions throughout the study were run in duplicate and samples that failed to amplify both times were discarded as negative. Gene expression data were analyzed using a generalized mixed model with a random factor ‘Family’ nested with *Wolbachia* × DENV load, with both ‘*Wolbachia*’ and ‘DENV load’ set as fixed factors. Statistics were performed using IBM SPSS Statistics (v.23) and GraphPad Prism 6.

## Additional file

Additional file 1: Table S1. Primers and probes used for qPCR gene detection. Gene IDs taken from VectorBase and UniProt. **Figure S1.** Head DENV loads correlate with Carcass DENV loads. DENV loads in the head were directly correlated to the same individual’s DENV loads in the carcass using Pearson’s correlation. Each dot depicts an individual either WT (blue, filled circles) or *w*Mel-infected (green, filled squares). **Figure S2.** Head DENV loads negatively correlate with *Wolbachia* loads. Individual DENV loads were directly correlated to the same individual’s *Wolbachia* loads using Pearson’s correlation. Each square depicts an individual. **Figure S3.** *Wolbachia* loads after viral injection. No significant differences in *Wolbachia* levels were observed between media-injected mosquitoes (black) and virus-injected mosquitoes (green). **Figure S4.** Interaction plots on the expression of the four tested genes. Parallel lines show no-interaction occurring between main effects DENV Load (High/Low) and *Wolbachia* (+/−) (**a**, **c**). Non-parallel lines show strong interaction (**b**, **d**). (PDF 512 kb)
