# Supplementary material for: Family level variation in Wolbachia-mediated dengue virus blocking in Aedes aegypti
Source: Parasit Vectors. 2017 Dec 28;10:622. doi: 10.1186/s13071-017-2589-3 (PMC5746003; doi:10.1186/s13071-017-2589-3)
Supplement: Additional file 1: Table S1. — Primers and probes used for qPCR gene detection. Gene IDs taken from VectorBase and UniProt. Figure S1. Head DENV loads correlate with Carcass DENV loads. DENV loads in the head were directly correlated to the same individual’s DENV loads in the carcass using Pearson’s correlation. Each dot depicts an individual either WT (blue, filled circles) or wMel-infected (green, filled squares). Figure S2. Head DENV loads negatively correlate with Wolbachia loads. Individual DENV loads were directly correlated to the same individual’s Wolbachia loads using Pearson’s correlation. Each square depicts an individual. Figure S3. Wolbachia loads after viral injection. No significant differences in Wolbachia levels were observed between media-injected mosquitoes (black) and virus-injected mosquitoes (green). Figure S4. Interaction plots on the expression of the four tested genes. Parallel lines show no-interaction occurring between main effects DENV Load (High/Low) and Wolbachia (+/−) (a, c). Non-parallel lines show strong interaction (b, d). (PDF 512 kb) [file 13071_2017_2589_MOESM1_ESM.pdf]

Table S1. – Primers and probes used for qPCR gene detection. Gene IDs taken from VectorBase and UniProt.

Gene ID	*Aedes* gene name	Direction	Sequence (5'-3')	Gene function
AAEL000718	*vir-1*	Fw	GCCAAAGTCCGGTATTCTTC	Antiviral effector
		Rv	TTCACGAGATCGTCAAGGTAA	
AAEL017251	*argonaute-2*	Fw	CCGTTCTGGACATGACTTGC	Catalytic compound of RISC, cleavage of mRNA
		Rv	CACAGCTCATGGTTGCTTCC	
AY190283.1	*Sterol Carrier Protein 2*	Fw	AAGTTTTCGCCAAGATCGCT	Non-specific lipid absorption and mobilization
		Rv	TTCTTGACAACCTTGCCACC	
AAEL009745	*Nitric oxide synthase*	Fw	AGGTTCGCAGTGTTTGCTCT	Nitric oxide synthesis / immune signalling
		Rv	GGTGCCCATTTCCTAAAAGC	
AAEL004175	*RpS17*	Fw	TCCGTGGTATCTCCATCAAGCT	Ribosomal small subunit assembly
		Rv	CACTTCCGGCACGTAGTTGTC	
		Pr	FAM-CAGGAGGAGGAACGTGAGCGCAG-BHQ1	
NC_001474.2	DENV	Fw	AAGGACTAGAGGTTAGAGGAGACCC	
		Rv	CGTTCTGTGCCTGGAATGATG	
		Pr	FAM-AACAGCATATTGACGCTGGGAGAGACCAGA-BHQ1	
AE017196	*Wolbachia*	Fw	GTATCCAACAGATCTAAGC	*w*Mel IS5 repeat
		Rv	ATAACCCTACTCATAGCTAG	
		Pr	LC640-TGAAATGGAAAAATTGGCGAGGTGTAGG-BHQ2	

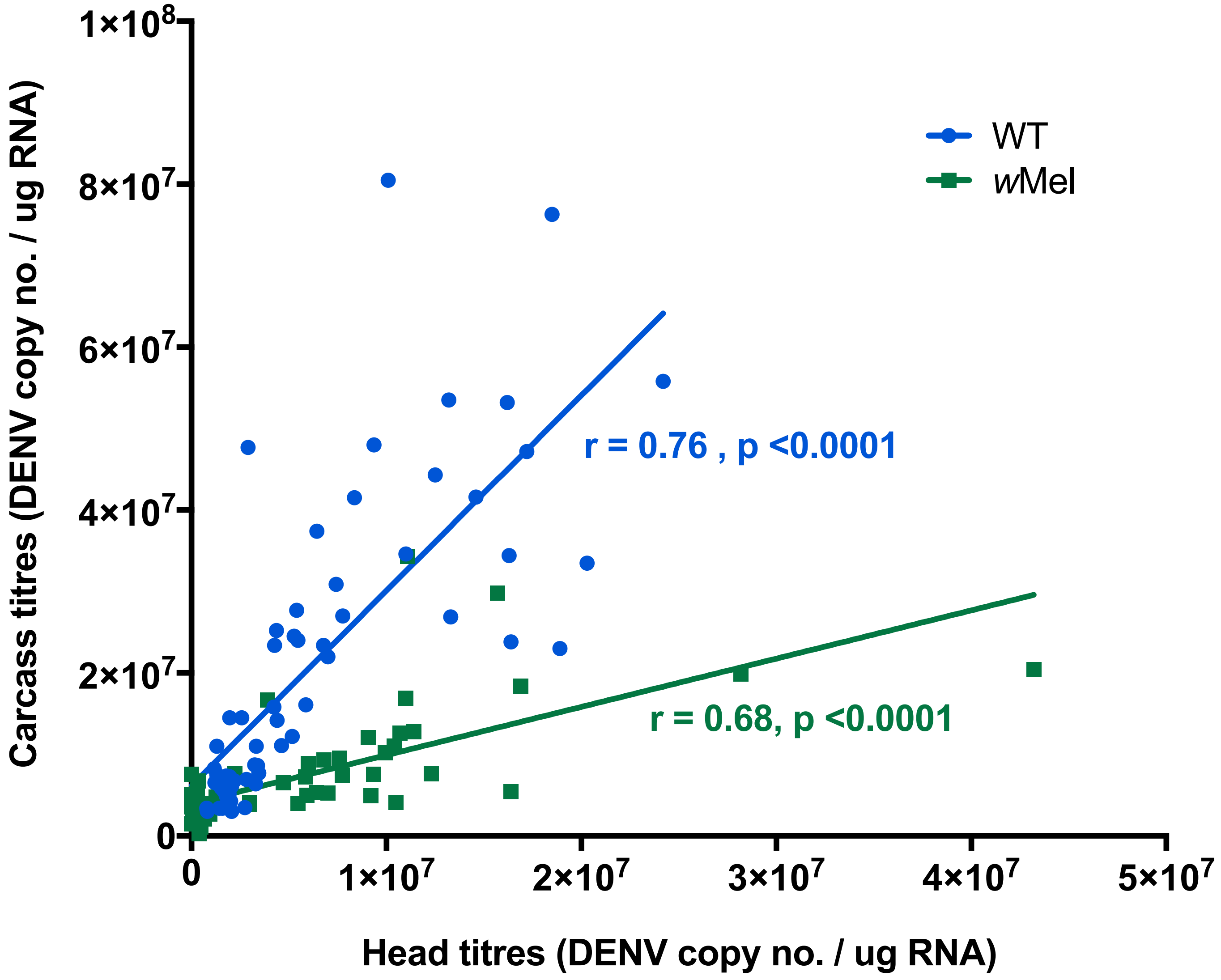

WT
wMel
r = 0.76 , p <0.0001
r = 0.68, p <0.0001
Carcass titres (DENV copy no. / ug RNA)
Head titres (DENV copy no. / ug RNA)
1×10^8
8×10^7
6×10^7
4×10^7
2×10^7
0
1×10^7
2×10^7
3×10^7
4×10^7
5×10^7

FIGURE S1

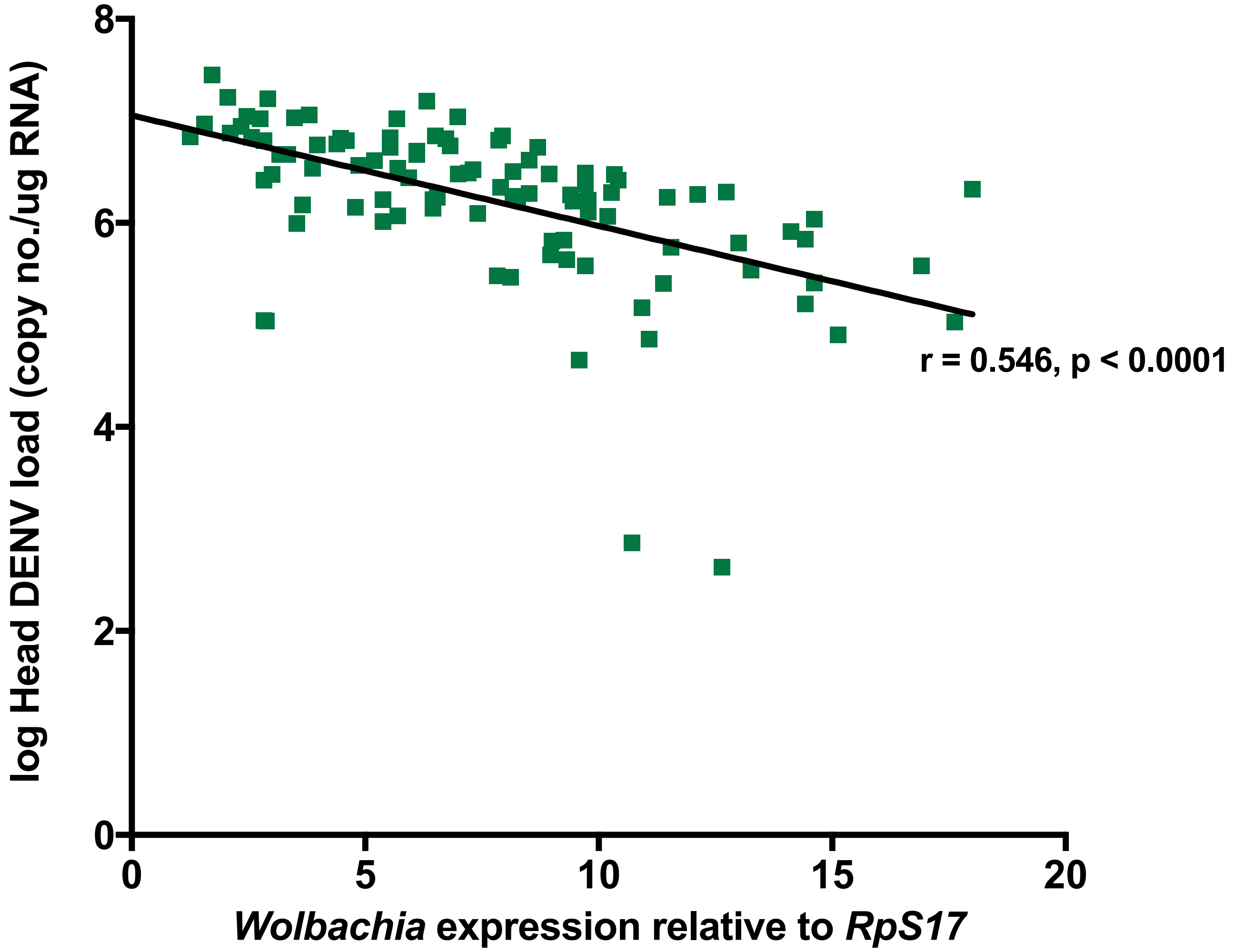
8
6
4
2
0
0
5
10
15
20
log Head DENV load (copy no./ug RNA)
Wolbachia expression relative to *RpS17*
r = 0.546, p < 0.0001

FIGURE S2

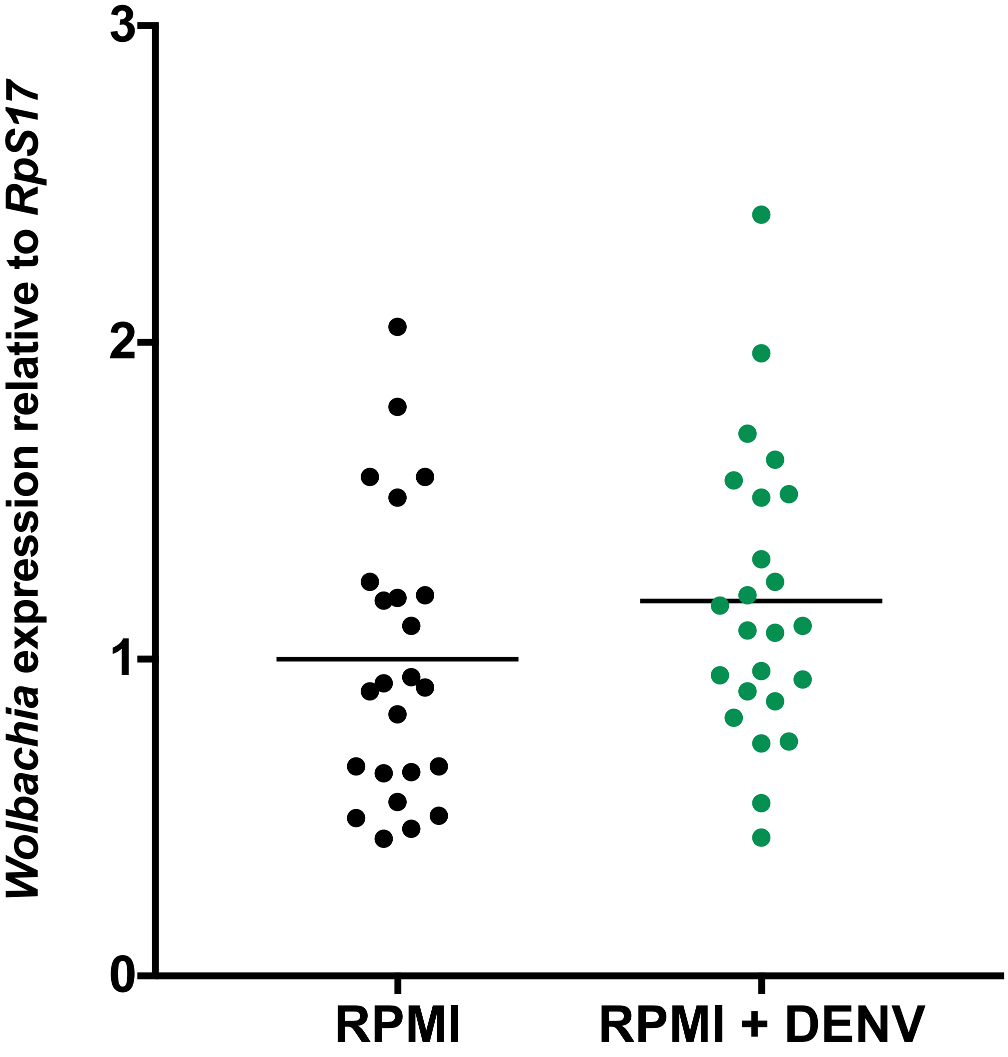

3
2
1
0
Wolbachia expression relative to RpS17
RPMI
RPMI + DENV

FIGURE S3

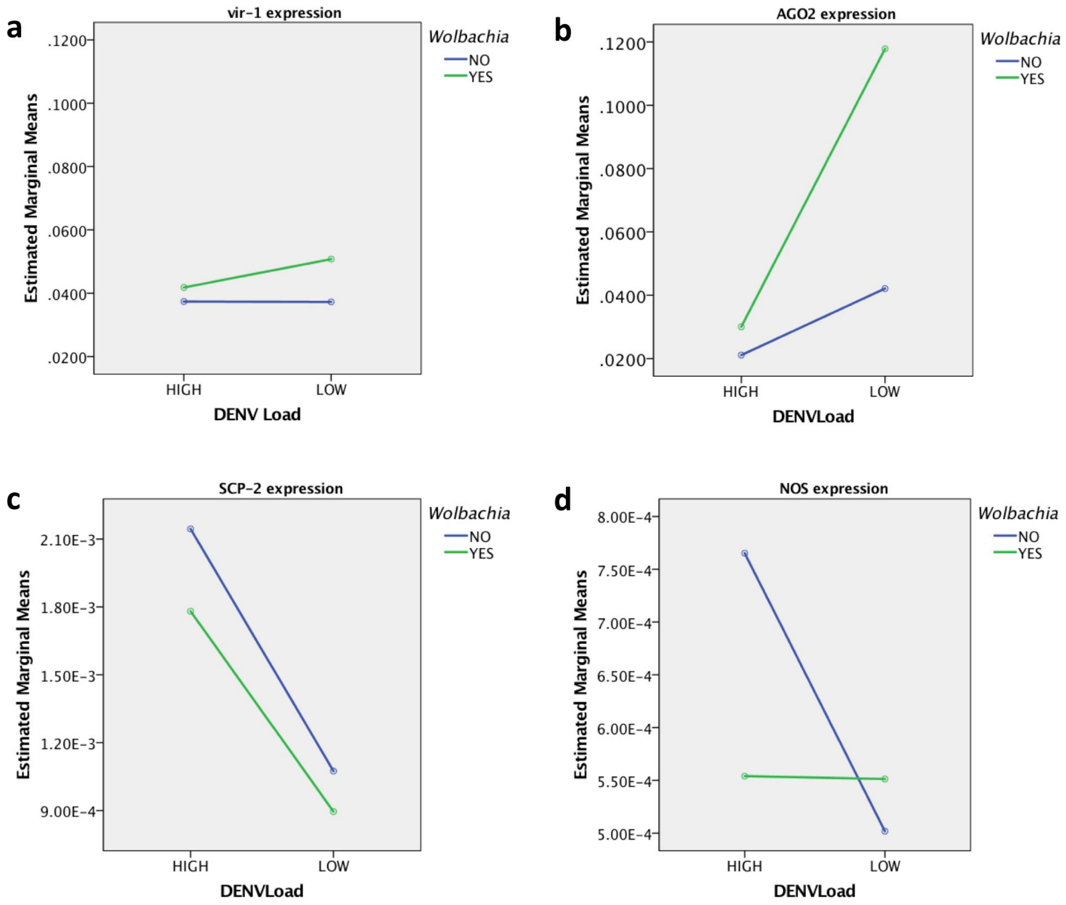

a
vir-1 expression
Wolbachia
NO
YES
Estimated Marginal Means
.1200
.1000
.0800
.0600
.0400
.0200
HIGH
LOW
DENV Load
b
AGO2 expression
Wolbachia
NO
YES
Estimated Marginal Means
.1200
.1000
.0800
.0600
.0400
.0200
HIGH
LOW
DENVLoad
c
SCP-2 expression
Wolbachia
NO
YES
Estimated Marginal Means
2.10E-3
1.80E-3
1.50E-3
1.20E-3
9.00E-4
HIGH
LOW
DENVLoad
d
NOS expression
Wolbachia
NO
YES
Estimated Marginal Means
8.00E-4
7.50E-4
7.00E-4
6.50E-4
6.00E-4
5.50E-4
5.00E-4
HIGH
LOW
DENVLoad

FIGURE S4
